# Family history and the risk of gastric cancer

**DOI:** 10.1038/sj.bjc.6605380

**Published:** 2009-11-03

**Authors:** M Yaghoobi, R Bijarchi, S A Narod

**Affiliations:** 1 Department of Clinical and Metabolic Genetics, Hospital for Sick Children, University of Toronto, Toronto, Ontario, Canada; 2 Women's College Research Institute, Women's College Hospital and the University of Toronto, Toronto, Ontario, Canada; 3 Division of Respirology, Department of Medicine, St Michael's Hospital, University of Toronto, Toronto, Ontario, Canada

**Keywords:** gastric cancer, hereditary, polymorphism, familial

## Abstract

Both environmental and genetic factors have a role in the aetiology of gastric cancer. The nature of the genetic factors has not been well-studied and, outside of a few rare cancer syndromes, the genes involved have not been identified. Having a first-degree relative with gastric cancer is a consistent risk factor for gastric cancer, although the magnitude of the odds ratio (OR) associated with a positive family history varies with the ethnic group and with the geographic region. In published case–control studies, the odds ratio varies from approximately 2 to 10, depending on the country. Unlike other common adult cancers, the risk of gastric cancer in migrants is similar to that of the population of origin and does not approach that of the host population in the first generation post-migration. It is hoped that molecular studies, including genomewide association studies (GWAS), will illuminate the genetic factors underlying this important association.

Gastric cancer is the fourth most common cancer worldwide and is the second most frequent cause of death from cancer (Parkin, 2004). Each year, approximately 700 000 people die of gastric cancer, representing about 10% of all cancer deaths (Parkin *et al*, 2001). Gastric cancers have been subdivided on the basis of their histological appearance into diffuse and intestinal types, and into cardia and non-cardia sites on the basis of their location (Lauren, 1965). The diffuse type appears, on average, at an earlier age than the intestinal type (Eto *et al*, 2006). It is believed that both environmental and genetic factors have causative roles, and it is possible that the risk factors are different for the various subtypes. The most important environmental risk factor is infection with *Helicobacter Pylori*, in particular for the non-cardia type of gastric cancer (Helicobacter and Cancer Collaborative Group, 2001). Suggested dietary risk factors include an increased intake of nitrites, nitrosamines and of salted foods and a low intake of fruits and vegetables (Jakszyn and González, 2006; Tsugane and Sasazuki, 2007). Smoking is also a risk factor (Neugut *et al*, 1996). The roles of familial and genetic factors have not been well-studied. Approaches to the study of genetic factors include case–control studies, molecular studies of cancer susceptibility and family-based studies. The key studies in the field are reviewed below.

## Worldwide distribution of gastric cancer

**Figure 1:**
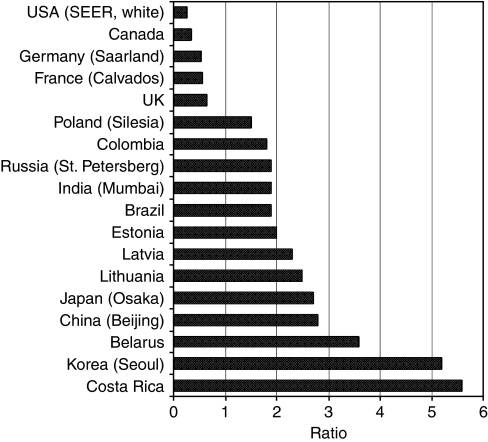
The ratio of gastric cancer to colon cancer in selected countries.

The international distribution of gastric cancer differs markedly from that of most other common adult tumours (e.g., breast, prostate and colon) in that the worldwide trend in incidence does not seem to parallel trends in westernization; that is, the rates across populations do not seem to be associated with increasing BMI, with a high-fat diet or with a sedentary lifestyle. In most western countries, the risk of (non-cardia) gastric cancer has declined steadily over the past 50 years, but the risk of cardia gastric cancer is stable, or is rising. In addition, the regional variation in the incidence of gastric cancer is also remarkable, which is much more pronounced than that of other common cancers; for example, in China, the annual adjusted rate is 20 per 100 000 for men in Beijing, compared with 145 for men in the Changle province (Cancer Incidence in Five Continents, 2002). Again, within a given geographic region, there is marked variation according to the ethnic group – this is not observed to the same extent for other common cancers. For example, in Los Angeles, the rates of stomach cancer vary from 7 to 43 per 100 000 per year by ethnic group, whereas, in the same city, the ethnic-specific rates for colon cancer vary modestly – from 17 to 28 per 100 000 per year (Table 1 and Figure 1) (Cancer Incidence in Five Continents, 2002). The reason for the extensive variation is unknown, and may include environmental exposures, but is also consistent with an important genetic contribution (however, the decline in incidence in western populations argues against a purely genetic aetiology – likely both factors will prove to be important).

## Familial risk

Familial relative risk is a hallmark of genetic susceptibility. Theoretically, familial clustering may also be because of non-hereditary factors, such as exposure to *H. Pylori*, smoking or to a common diet. It is also possible that a gene/environment interaction is involved – this would be the case when genetic susceptibility to infection or to the mutagenic effects of smoking (or another carcinogen) were responsible for the observed familial association.

The importance of family history as a risk factor for gastric cancer is most readily evaluated using the case–control approach. We reviewed 15 case–control studies of family history and gastric cancer (Table 2); six studies were from Europe, seven from East Asia, one from India and one from the United States. In these studies, the odds ratios (OR) of gastric cancer were calculated with reference to one or more first- or second-degree relatives with gastric cancer, but the definition of a positive family history varied between studies. Most studies combined all first-degree relatives (i.e., siblings and parents) but one study published results separately for siblings and parents (Bakir *et al*, 2000, 2003).

Dhillon *et al* (2001) studied 695 cases and 629 population-based controls from the United States and estimated the familial relative risk for gastric cancer to be 2.2 (95% CI 1.5–3.3). They adjusted for age, gender, smoking, alcohol intake, BMI and household income, but not for *H. pylori* infection. The risk was stronger for individuals reporting two or more relatives with gastric cancer (OR=12.1; 95% CI 1.4–108).

Seven European studies were reviewed, two each from Turkey and Italy and one each from Poland, Germany and Spain.

In a population-based case–control study of 464 gastric cancer patients and 480 controls from Warsaw, Poland, a three-fold increase in risk was associated with a history of gastric cancer in a first-degree relative (OR=3.5; 95% Cl 2.0–6.2) (see Lissowska *et al*, 1999). No excess risk was observed with any other form of cancer.

In the large Turkish study, the results for siblings were published in 2000 (see Bakir *et al*, 2000) and the results for parents were published in 2003 (see Bakir *et al*, 2003). This large study included 1240 cases of gastric cancer and 1240 hospital-based non-cancer controls. In that study, 14% of the cases reported a sibling with gastric cancer (168 out of 1240) and 12% of the cases reported a parent with gastric cancer (148 out of 1240). The corresponding OR were 10.1 for siblings (95% CI 6.1–16.8) and 6.6 for parents (95% CI 4.2–10.4). The results were not adjusted for other environmental factors.

In Italy, Palli *et al* (2001) estimated the OR to be 1.8 (95% CI 1.6–2.0), based on 126 cases of gastric cancer and 561 community-based healthy controls. La Vecchia *et al* (1992) also studied Italian patients, and estimated the familial relative risk to be 2.6 (95% CI 1.9–3.4), based on 628 cases and 1776 hospital-based controls.

A small study from Germany (Brenner *et al*, 2000) showed a relative risk of 2.9 (95% CI 1.3–6.5). In this study, Brenner *et al* showed that a family history of gastric cancer was also associated with an increased prevalence of *H. pylori* infection. Individuals with both *H. pylori* infection and a positive family history of gastric cancer faced an eight-fold increased risk of development of gastric cancer, compared with people with neither risk factor. However, after adjustment for *H pylori*, the OR for the development of gastric cancer, given a positive family history, was 2.8, indicating that the familial and infectious risk factors are independent.

A study from Spain with 404 cases and 404 controls reported an OR of 3.4 (OR=3.4; 95% CI 1.9–6.0) (García-González *et al*, 2007).

In general, the rates of gastric cancer in Asia are higher than the rates in Europe or North America, but there is marked variation within the continent. The highest reported rates are from Japan and Korea; in these countries, the rates are typically about 70 per 100 000 per year (Cancer Incidence in Five Continents, 2002). In contrast, India has one of the lowest reported rates (Cancer Incidence in Five Continents, 2002). Of the eight identified case–control studies from Asia, the highest reported OR was from Korea (RR=9.9; 95% CI 6.5–15), but this study was relatively small (238 cases and 108 hospital-based controls) (Hong *et al*, 2006). Remarkably, 94 of 108 Korean patients (87%) with gastric cancer reported a first-degree relative with gastric cancer – this is a much higher proportion than has been reported elsewhere.

Five studies from different regions of Japan reported OR ranging from 1.5 to 3.5. The highest OR was reported by Eto *et al* (2006). The OR was 3.5 (95% CI 3.3–3.8) after adjustment for age and gender. In this study, the overall OR were similar for the intestinal and diffuse subtypes, but among patients diagnosed with gastric cancer before age 43, the OR for a family history with the intestinal type was 12.5 (95% CI 4.8–32). The lowest reported OR in Japan was 1.5 (95% CI 1.2–1.8), based on a large study on 614 cases and 2444 hospital-based controls by Minami *et al* (2003). Ikeguchi *et al* (2001) studied 2025 cases of gastric cancer and 926 hospital-based controls. The familial OR was approximately 2 (RR=1.9; 1.6–2.2). In this study, 14 of the 926 cases (1.5%) were from a family with four or more cases of gastric cancer, suggesting that a dominant gene may be responsible for a small proportion of cases. Huang *et al* (1999) studied 887 cases and 28 619 non-gastric cancer controls. They also estimated the OR to be 1.9 (95% CI 1.6–2.1). The OR was adjusted for age, sex, smoking, alcohol, consumption of salty foods, and fruit and vegetable intake. Nagase *et al* (1996) studied 136 cases and 136 controls and reported a crude OR of 2.7 (95% CI 1.7–4.4). In this study, family history was a stronger risk factor for females than for males (OR=4.5 for females and 1.2 for males).

Chen *et al* (2004) studied 176 cases of cancer of the gastric cardia and 579 hospital-based controls from Taiwan. They reported a relative risk of 2.5 (95% CI 1.3–4.8), after adjusting for age, sex, ethnicity, education, BMI, marital status and socioeconomic status. It is not known whether the cancers in the affected relatives were also in the gastric cardia.

A study from India included 388 cases and 388 non-gastric cancer controls. The OR was 5.7 (95% CI 1.3–26) (Gajalakshmi and Shanta, 1996), but this was based on a small number of familial cases (*n*=12) and controls (*n*=2).

In summary, the case–control studies reviewed in this study consistently report that a family history of gastric cancer is a risk factor for gastric cancer. There were no negative studies. In the majority of studies, the risk ratio was between 1.5-fold and 3.5-fold, but studies from Korea, Turkey and India reported higher OR.

It is possible that familial clustering of non-genetic risk factors, such as *H Pylori* infection or a diet high in salty foods and/or low in fruit and vegetables, could contribute to familial clustering. However, in the studies that adjusted for one or more of these risk factors, the adjustment did not attenuate the relative risk associated with a positive family history. This argues in favour of genetic susceptibility underlying the observed familial clustering. The relevant genes are so far unknown.

A correlation between the national incidence rate and the familial relative risk could be because of international variation in the prevalence of one or more alleles of a susceptibility gene. It is of interest that of the three countries with the highest reported familial relative risks, one (Korea) has a very high incidence of gastric cancer, one (India) has a low incidence of gastric cancer) and one (Turkey) has an intermediate rate (Table 3).

Furthermore, even when the relative risk of gastric cancer were found to be elevated to a similar extent for first-degree relatives of the patients in the different studies, the actual risk to relatives will depend on the baseline rate, which in turn depends on the ethnic group and the country of residence. For example, the lifetime risk of gastric cancer is approximately 1% in Canada and the United States, but in much of Japan, the lifetime risk of gastric cancer in men exceeds 10%. In Canada, a male with a first-degree relative with gastric cancer, given a relative risk of 2.9, will face a lifetime risk of about 3% (Cancer Incidence in Five Continents, 2002). In contrast, when a relative risk of 2.9 is applied to relatives of patients in Japan, the lifetime risk may approach 30%. In the studies reviewed here, the countries with the highest familial relative risks were Turkey, India and Korea, whereas the countries with the highest baseline risks were Japan and Korea. Other countries with high incidences of gastric cancer include Colombia, Costa Rica, Russia, Belarus and the Baltic Republics (Lithuania, Latvia and Estonia) (Table 3). To our knowledge, case–control studies have not been conducted in Latin America or in Eastern Europe.

## Gastric cancer syndromes

Gastric cancer is an infrequent component of several inherited cancer predisposition syndromes, including hereditary nonpolyposis colon cancer, familial adenomatous polyposis and Peutz-Jeghers syndrome (Giardiello *et al*, 1987; Offerhaus *et al*, 1992; Watson and Lynch, 1993; Lynch and Smyrk, 1996). Hereditary diffuse gastric cancer (HDGC) is a rare, autosomal dominant inherited form of gastric cancer. Germline CDH1 mutations were first described in three New Zealand families with early-onset, poorly differentiated, high-grade, diffuse gastric cancer (Guilford *et al*, 1998). In a recent international study of 38 families with HDGC, 15 CDH1 mutations were found, representing 39% of the families tested (Kaurah *et al*, 2007). The penetrance for gastric cancer among individuals with a CDH1 mutation is estimated to be about 40%. However, considering the rarity of the syndrome, CDH1 mutations make a small contribution to the total burden of familial gastric cancer.

The genetic abnormalities that predispose to gastric cancer seem not to be the same in all countries – possibly because of different frequencies of the predisposing mutations or the need for environmental co-factors. There are several examples. Gastric cancer is not a classical component of the breast–ovarian cancer syndrome, but in Poland, BRCA2 germline mutations were identified in 21% of families with both gastric and breast cancer (Jakubowska *et al*, 2002). In Japan, gastric cancer is an expression of familial polyposis coli, whereas in North America, colon cancers are dominant (Shimoyama *et al*, 2004). Similarly, gastric cancer is not a central component of the Li-Fraumeni syndrome in North America, but germline mutations in p53 have been reported in three gastric cancer families from Japan (Yamada *et al*, 2007). Two other studies searched for germline p53 mutations in 31 gastric cancer families from Portugal and in 35 gastric cancer families from Germany. One germline p53 mutation was found in each of these series (Keller *et al*, 2004; Oliveira *et al*, 2004).

Carriers of germline mutations in the mismatch repair (MMR) genes, MSH2 and MLH1, also have an increased risk of gastric cancer (Imai and Yamamoto, 2008). Typically, germline mutations in the MMR genes are the cause of hereditary non-polyposis colon cancer (HNPCC), but somatic (i.e., cancer specific) mutations in these genes are also common in non-familial cases of colon cancer. Germline or somatic mutations in mismatch repair genes usually result in the presence of microsatellite instability (MSI) in the colon tumour tissue. Although MSI is present in 20 to 30% of cases of gastric cancer (Renault *et al*, 1996), germline or somatic mutations in the mismatch repair genes are rarely observed in MSI-positive cases (Keller *et al*, 1996). In one study, MSI was significantly associated with antral tumours of the stomach and a positive family history of gastric cancer (Ottini *et al*, 1997).

## Polymorphic variants

Currently, there is much interest in the identification of low-penetrance alleles that are associated with an increased risk for common forms of cancer. It is believed that for many adult cancers, familial aggregation may be more the result of a number of low-penetrant alleles acting in combination, rather than one or a few highly penetrant dominant cancer genes. Until recently, gastric cancer has not been well-studied in this regard, but over the past few years, several investigators have begun to evaluate candidate genes. Genes have been selected either because they are believed to influence susceptibility to *H. Pylori* infection, to limit the production of gastric acid, or moderate local inflammation or influence the metabolism of potential carcinogens. To date, no consistent association has emerged and only a few of the key studies are reviewed here.

A series of studies has been conducted on a sample of Polish gastric patients and controls; these focus largely on the cytokines and related proteins that are believed to be involved in inflammatory processes. Interleukin 1B (IL1B) is pro-inflammatory and is an inhibitor of gastric acid secretion. El-Omar *et al* studied a non-coding polymorphism in IL1B; they found that individuals who carried two T alleles at this locus faced a 2.6-fold increase in the risk of gastric cancer (95% CI 1.7–3.9) (El-Omar *et al*, 2000). A related gene (IL1RN) from the same chromosome region is also polymorphic; the researchers reported a 3.7-fold risk (95% CI 2.4–5.7) associated with a specific genotype of a VNTR polymorphism in the gene (El-Omar *et al*, 2000). A polymorphic variant in the promoter of mannose-binding lectin-2 was also associated with a 1.8-fold increase in the risk of gastric cancer in Poland (95% CI 1.1–2.9) (Baccarelli *et al*, 2006).

In a study of gastric cancer patients from Mexico, the IL10-592 CC genotype was associated with an OR of 2.2 (95% CI 1.0–4.6) for intestinal-type gastric cancer (Sicinschi *et al*, 2006). A later Spanish study, which included most of these cytokine variants, did not confirm the reported associations (García-González *et al*, 2007). Wang *et al* conducted a meta-analysis of 39 studies, which included 6863 cases and 8434 controls (Wang *et al*, 2007). The summary OR of gastric cancer risk associated with the IL-1B-511T polymorphism was 1.26 (95% CI 1.03–1.55) and the summary OR associated with the IL-1RN^*^2 allele was 1.20 (95% CI 1.01–1.41). The IL-1B-511T polymorphism was associated with an increased risk of gastric cancer of the intestinal type (OR=1.76, 95% CI 1.12–2.57). The IL-1RN^*^2 variant was associated with an increased risk of gastric cancer among Caucasians (OR=1.30, 95% CI 1.09–1.54).

In summary, the studies to date in aggregate do not support the hypothesis that familial aggregation of gastric cancer can be accounted for by low-penetrance polymorphic variants. However, association studies to date have been small and few ethnic groups have been represented. It is possible that the susceptibility variants are different for different populations, due to variability in modifier genes or environmental factors. It is therefore important that large-scale international studies and/or meta-analyses be conducted to see whether there are consistent genetic associations or whether ethnic-specific effects are present. It is expected that these will soon be extended to include genomewide association studies (GWAS).

## Screening and prevention

It is hoped that the identification of genetic factors for gastric cancer will lead to the development of a clinical test that can be used to identify individuals at high risk. This classification may allow us to focus preventive efforts and screening programs on individuals at high risk. The exact means of prevention may depend on the susceptibility gene involved (and the molecular pathway to be targeted). It may be that different genes operate in different high-risk populations.

Aspirin use has been associated with a modest reduction in the risk of gastric cancer. The use of aspirin (HR, 95% CI 0.64, 0.47–0.86) or other NSAIDs (0.68, 0.51–0.92) was associated with a significantly lower risk of gastric non-cardia cancer (Abnet *et al*, 2009). It is important to ask whether proton pump inhibitors, which are prescribed to reduce gastric acid protection, can potentially be used for chemoprevention of gastric cancer in high-risk individuals.

It is hoped that the eradication of *H Pylori* infection through antibiotic therapy will reduce the incidence of gastric cancer. A randomised trial of antibiotic therapy was conducted in a cohort of patients with resectable gastric cancer, with the aim of preventing second primary cancers (Fukase *et al*, 2008). After 3 years of follow-up, a second gastric carcinoma developed in nine patients in the treatment arm and in 24 patients in the control group (OR=0.35, 95% CI 0.16–0.78; *P*=0.009). It may be that in high-risk areas all residents should be screened for *H. Pylori* infection and offered eradication therapy. However, in low-risk areas, therapy might be targeted to high-risk individuals, such as those who are genetically predisposed.

To date, with the exception of diffuse familial gastric cancer (Lynch *et al*, 2008), genetic markers cannot be used to identify individuals at high risk of gastric cancer. Similarly, preventive surgery is limited to those with a mutation in E-cadherin, which predisposes to hereditary diffuse gastric cancer (Lynch *et al*, 2008). The risk of gastric cancer for an individual can be estimated on the basis of family history, sex and place of residence. Currently, endoscopy screening is recommended only in high-risk areas such as Japan, and is based on residence and ethnic group rather than family history. It is possible that in the future, endoscopy might be offered to individuals in other countries who are determined to be at high risk, based on genetic markers.

## Conclusion

A positive family history is a strong and consistently reported risk factor for gastric cancer, but the molecular basis for the familial aggregation is largely unknown. The known cancer syndromes do not account for a large part of the familial clustering. Unlike the situation for other common cancers, guidelines have not been developed for the assessment of the family history of individuals with gastric cancer. A recent systematic review of clinical patterns of genetic assessment and referral for 11 different types of cancer in the United Kingdom did not include gastric cancer (Featherstone *et al*, 2007). To some extent, this lack of attention may be because of the international distribution of gastric cancer cases; that is, high rates in Asia, Eastern Europe and Latin America, but relatively low rates in North America and Europe. There may also be a lack of awareness of the extent to which gastric cancer is familial. In most studies, the familial relative risk is approximately three-fold, which is larger than that observed for most other adult forms of solid cancer, with the exception of ovarian cancer. In India, Korea and Turkey, much higher relative risks have been reported. It will be important to confirm these results and to conduct studies in other regions of high incidence. Molecular epidemiology studies, such as GWAS, may prove to be useful in identifying the genetic factors responsible.

## Figures and Tables

**Table 1:** Rates of stomach and colon cancer in Los Angeles (males, per 100 000 per year)

	**Stomach**	**Colon**	**Ratio**
Non-Hispanic white	7.3	24.5	0.30
Hispanic white	14.4	21.0	0.69
Black	11.1	29.6	0.38
Chinese	14.5	19.9	0.73
Filipino	7.4	17.2	0.43
Japanese	21.8	27.7	0.79
Korean	43.4	20.0	2.17

Source: Cancer incidence in Five Continents, volume VIII.

**Table 2:** Case–control studies of gastric cancer and family history

			**Case group**		**Control group**		
**Study**	**Country**	**Family history**	***n***	***N***	***n***	***N***	**RR (95% CI)**
(Lissowska *et al*, 1999)	Poland	First-degree relative		464		480	3.5 (2.0–6.2)
(Brenner *et al*, 2000)	Germany		10	68 (15%)	12	239	2.9 (1.3–6.5)
(La Vecchia *et al*, 1992)	Italy	First-degree relatives	79	628 (13%)	87	1776	2.6 (1.9–3.4)
(Palli *et al*, 2001)	Italy	First-degree relatives	40	126 (32%)	74	561	1.8 (1.6–2.0)
(García-González *et al*, 2007)	Spain	One first-degree or two second-degree relatives	51	290 (18%)	17	286	3.0 (1.8–5.0)
(Bakir *et al*, 2000)	Turkey	Siblings	168	1240 (14%)	19	1240	10.1 (6.1–16.8
(Bakir *et al*, 2003)	Turkey	Parents	148	1240 (12%)	25	1240	6.6 (4.2–10.4)
(Dhillon *et al*, 2001)	USA	First-degree relative	70	629 (11%)	35	695	2.2 (1.5–3.3)
(Gajalakshmi and Shanta, 1996)	India	Undefined	12	388 (3.5%)	2	388	5.7 (1.3–26)
(Minami and Tateno, 2003)	Japan	First-degree relatives	140	614 (23%)	369	2444	1.5 (1.3–1.8)
(Huang *et al*, 1999)	Japan	First-degree relatives	207	887 (23%)	3608	28 619	1.9 (1.6–2.1)
(Ikeguchi *et al*, 2001)	Japan	First-, second- and third-degree relatives	216	926 (23%)	254	2025	1.9 (1.6–2.2)
(Nagase *et al*, 1996)	Japan		49	136 (365)	28	136	2.7 (1.7–4.4)
(Eto *et al*, 2006)	Japan	First-degree relative	543	1400 (39%)	1475	13 467	3.5 (3.3–3.8)
(Hong *et al*, 2006)	Korea	First-degree relatives	94	108 (87%)	21	238	9.9 (6.5–15)
(Chen *et al*, 2004)	Taiwan	First-degree relatives	47	176 (27%)	54	579	2.5 (1.3–4.8)

**Table 3:** Estimated rates of gastric cancer and colon cancer in selected countries

	**Age-standardised rate (males) per 100 000 per year**	
**Country**	**Gastric**	**Colon**
United States (SEER, white)	6.6	25.5
Canada	9.1	25.9
United Kingdom	13.1	20.6
France (Calvados)	12.2	22.3
Germany (Saarland)	14.7	27.3
Russia (St Petersburg)	38.3	19.9
Poland (Silesia)	22.9	15.6
Estonia	31.9	16.1
Latvia	28.2	12.5
Lithuania	29.7	11.8
Belarus	40.5	11.1
Turkey	12.2	
Japan (Osaka)	59.9	22.1
Korea (Seoul)	68.0	13.1
China (Beijing)	19.8	7.2
India (Mumbai)	6.3	3.4
Colombia	18.8	10.4
Costa rica	40.1	7.1
Brazil	21.2	11.3

Source: Cancer Incidence in Five Continents, Globocan.

## References

[bib1] Abnet CC, Freedman ND, Kamangar F, Leitzmann MF, Hollenbeck AR, Schatzkin A (2009) Non-steroidal anti-inflammatory drugs and risk of gastric and oesophageal adenocarcinomas: results from a cohort study and a meta-analysis. Br J Cancer 100(3): 551–557 19156150 10.1038/sj.bjc.6604880 PMC2658549

[bib2] Baccarelli A, Hou L, Chen J, Lissowska J, El-Omar EM, Grillo P, Giacomini SM, Yaeger M, Bernig T, Zatonski W, Fraumeni Jr JF, Chanock SJ, Chow WH (2006) Mannose-binding lectin-2 genetic variation and stomach cancer risk. Int J Cancer 119(8): 1970–1975 16721783 10.1002/ijc.22075

[bib3] Bakir T, Can G, Erkul S, Siviloglu C (2000) Stomach cancer history in the siblings of patients with gastric carcinoma. Eur J Cancer Prev 9: 401–408 11201678 10.1097/00008469-200012000-00005

[bib4] Bakir T, Can G, Siviloglu C, Erkul S (2003) Gastric cancer and other organ cancer history in the parents of patients with gastric cancer. Eur J Cancer Prev 12: 183–189 12771555 10.1097/00008469-200306000-00003

[bib5] Brenner H, Arndt V, Stürmer T, Stegmaier C, Ziegler H, Dhom G (2000) Individual and joint contribution of family history and *Helicobacter pylori* infection to the risk of gastric carcinoma. Cancer 88: 274–279 10640957 10.1002/(sici)1097-0142(20000115)88:2<274::aid-cncr5>3.0.co;2-9

[bib6] Cancer Incidence in Five Continents (2002) Vol. VIII. IARC Press: Lyon

[bib7] Chen MJ, Wu DC, Ko YC, Chiou YY (2004) Personal history and family history as a predictor of gastric cardiac adenocarcinoma risk: a case-control study in Taiwan. Am J Gastroenterol 99: 1250–1257 15233662 10.1111/j.1572-0241.2004.30872.x

[bib8] Dhillon PK, Farrow DC, Vaughan TL, Chow WH, Risch HA, Gammon MD, Mayne ST, Stanford JL, Schoenberg JB, Ahsan H, Dubrow R, West AB, Rotterdam H, Blot WJ, Fraumeni Jr JF (2001) Family history of cancer and risk of esophageal and gastric cancers in the United States. Int J Cancer 93: 148–152 11391635 10.1002/ijc.1294

[bib9] El-Omar EM, Carrington M, Chow WH, McColl KEL, Bream JH, Young HA, Herrera J, Lissowska J, Yuan CC, Rothman N, Lanyon G, Martin M, Fraumeni Jr JF, Rabkin CS (2000) Interleukin-1 polymorphisms associated with increased risk of gastric cancer. Nature 404: 398–402 10746728 10.1038/35006081

[bib10] Eto K, Ohyama S, Yamaguchi T, Wada T, Suzuki Y, Mitsumori N, Kashiwagi H, Anazawa S, Yanaga K, Urashima M (2006) Familial clustering in subgroups of gastric cancer stratified by histology, age group and location. Eur J Surg Oncol 32: 743–748 16762526 10.1016/j.ejso.2006.04.005

[bib11] Featherstone C, Colley A, Tucker K, Kirk J, Barton MB (2007) Estimating the referral rate for cancer genetic assessment from a systemic review of the evidence. Br J Cancer 10: 75–83 10.1038/sj.bjc.6603432 PMC2360013 17242707

[bib12] Fukase K, Kato M, Kikuchi S, Inoue K, Uemura N, Okamoto S, Terao S, Amagai K, Hayashi S, Asaka M, Japan Gast Study Group (2008) Effect of eradication of *Helicobacter pylori* on incidence of metachronous gastric carcinoma after endoscopic resection of early gastric cancer: an open-label, randomised controlled trial. Lancet 372(9636): 392–397 18675689 10.1016/S0140-6736(08)61159-9

[bib13] Gajalakshmi CK, Shanta V (1996) Lifestyle and risk of stomach cancer: a hospital-based case-control study. Int J Epidemiol 25: 1146–1153 9027518 10.1093/ije/25.6.1146

[bib14] García-González MA, Lanas A, Quintero E, Nicolás D, Parra-Blanco A, Strunk M, Benito R, Angel Simón M, Santolaria S, Sopeña F, Piazuelo E, Jiménez P, Pascual C, Mas E, Irún P, Espinel J, Campo R, Manzano M, Geijo F, Pellisé M, González-Huix F, Nieto M, Espinós J, Titó L, Bujanda L, Zaballa M, Spanish Gastroenterological Association AEG (2007) Spanish Gastroenterological Association AEG. Gastric cancer susceptibility is not linked to pro- and anti-inflammatory cytokine gene polymorphisms in whites: a nationwide multicenter study in Spain. Am J Gastroenterol 102: 1878–1892 17640324 10.1111/j.1572-0241.2007.01423.x

[bib15] Giardiello FM, Welsh SB, Hamilton SR, Offerhaus GJA, Gittelsohn AM, Booker SV, Krush AJ, Yardley JH, Luk GD (1987) Increased risk of cancer in the Peutz-Jeghers syndrome. New Engl J Med 316: 1511–1514 3587280 10.1056/NEJM198706113162404

[bib16] Guilford P, Hopkins J, Harraway J, McLeod M, McLeod N, Harawira P, Taite H, Scoular R, Miller A, Reeve AE (1998) E-cadherin germline mutations in familial gastric cancer. Nature 392: 402–405 9537325 10.1038/32918

[bib17] Helicobacter and Cancer Collaborative Group (2001) Gastric cancer and *Helicobacter pylori*: a combined analysis of 12 case control studies nested within prospective cohorts. Gut 49: 347–353 11511555 10.1136/gut.49.3.347 PMC1728434

[bib18] Hong SH, Kim JW, Kim HG, Park IK, Ryoo JW, Lee CH, Sohn YK, Lee JY (2006) Glutathione S-transferases (GSTM1, GSTT1 and GSTP1) and N-acetyltransferase 2 polymorphisms and the risk of gastric cancer. J Prev Med Pub Health 39: 135–140 16615268

[bib19] Huang X, Tajima K, Hamajima N, Inoue M, Takezaki T, Kuroishi T, Hirose K, Tominaga S, Xiang J, Tokudome S (1999) Effect of life styles on the risk of subsite-specific gastric cancer in those with and without family history. J Epidemiol 9: 40–45 10098352 10.2188/jea.9.40

[bib20] Ikeguchi M, Fukuda K, Oka S, Hisamitsu K, Katano K, Tsujitani S, Kaibara N (2001) Clinicopathological findings in patients with gastric adenocarcinoma with familial aggregation. Dig Surg 18: 439–443 11799292 10.1159/000050190

[bib21] Imai K, Yamamoto H (2008) Carcinogenesis and microsatellite instability: the interrelationship between genetics and epigenetics. Carcinogenesis 29: 673–680 17942460 10.1093/carcin/bgm228

[bib22] Jakszyn P, González CA (2006) Nitrosamine and related food intake and gastric and oesophageal cancer risk: a systematic review of the epidemiological evidence World. J Gastroenterol 12: 4296–4303 10.3748/wjg.v12.i27.4296 PMC4087738 16865769

[bib23] Jakubowska A, Nej K, Huzarski T, Scott RJ, Lubinski J (2002) BRCA2 gene mutations in families with aggregations of breast and stomach cancers. Br J Cancer 87: 888–891 12373604 10.1038/sj.bjc.6600562 PMC2376177

[bib24] Kaurah P, MacMillan A, Boyd N, Senz J, De Luca A, Chun N, Suriano G, Zaor S, Van Manen L, Gilpin C, Nikkel S, Connolly-Wilson M, Weissman S, Rubinstein WS, Sebold C, Greenstein R, Stroop J, Yim D, Panzini B, McKinnon W, Greenblatt M, Wirtzfeld D, Fontaine D, Coit D, Yoon S, Chung D, Lauwers G, Pizzuti A, Vaccaro C, Redal MA, Oliveira C, Tischkowitz M, Olschwang S, Gallinger S, Lynch H, Green J, Ford J, Pharoah P, Fernandez B, Huntsman D (2007) Founder and recurrent CDH1 mutations in families with hereditary diffuse gastric cancer. JAMA 297: 2360–2372 17545690 10.1001/jama.297.21.2360

[bib25] Keller G, Vogelsang H, Becker I, Plaschke S, Ott K, Suriano G, Mateus AR, Seruca R, Biedermann K, Huntsman D, Döring C, Holinski-Feder E, Neutzling A, Siewert JR, Höfler H. (2004) Germline mutations of the E-cadherin (CDH1) and TP53 genes, rather than of RUNX3 and HPP1, contribute to genetic predisposition in German gastric cancer patients. J Med Genet 41: e89 15173255 10.1136/jmg.2003.015594 PMC1735803

[bib26] Keller G, Grimm V, Vogelsang H, Bischoff P, Mueller J, Siewert JR, Hofler H (1996) Analysis for microsatellite instability and mutations of the DNA mismatch repair gene hMLH1 in familial gastric cancer. Int J Cancer 68: 571–576 8938136 10.1002/(SICI)1097-0215(19961127)68:5<571::AID-IJC3>3.0.CO;2-W

[bib27] La Vecchia C, Negri E, Franceschi S, Gentile A (1992) Family history and the risk of stomach and colorectal cancer. Cancer 70: 50–55 1606546 10.1002/1097-0142(19920701)70:1<50::aid-cncr2820700109>3.0.co;2-i

[bib28] Lauren P (1965) The two histological main types of gastric carcinoma: diffuse and so-called intestinal-type carcinoma. An attempt at a histo-clinical classification. Microbiol Scand 64: 31–49 10.1111/apm.1965.64.1.31 14320675

[bib29] Lissowska J, Groves FD, Sobin LH, Fraumeni Jr JF, Nasierowska-Guttmejer A, Radziszewski J, Regula J, Hsing AW, Zatonski W, Blot WJ, Chow WH (1999) Family history and risk of stomach cancer in Warsaw, Poland. Int J Cancer 8: 223–227 10.1097/00008469-199906000-00010 10443951

[bib30] Lynch HT, Smyrk T (1996) Hereditary nonpolyposis colorectal cancer (Lynch syndrome): an updated review. Cancer 78: 1149–1167 8826936 10.1002/(SICI)1097-0142(19960915)78:6<1149::AID-CNCR1>3.0.CO;2-5

[bib31] Lynch HT, Silva E, Wirtzfeld D, Hebbard P, Lynch J, Huntsman DG (2008) Hereditary diffuse gastric cancer: prophylactic surgical oncology implications. Surg Clin North Am 88: 759–778 18672140 10.1016/j.suc.2008.04.006 PMC2561947

[bib32] Minami Y, Tateno H (2003) Associations between cigarette smoking and the risk of four leading cancers in Miyagi Prefecture, Japan: a multi-site case-control study. Cancer Sci 94: 540–547 14529588 10.1111/j.1349-7006.2003.tb01480.x PMC11160141

[bib33] Nagase H, Ogino K, Yoshida I, Matsuda H, Yoshida M, Nakamura H, Dan S, Ishimaru M (1996) Family history-related risk of gastric cancer in Japan: a hospital-based case-control study. Jpn J Cancer Res 87: 1025–1028 8957058 10.1111/j.1349-7006.1996.tb03104.x PMC5920991

[bib34] Neugut AI, Hayek M, Howe G (1996) Epidemiology of gastric cancer. Semin Oncol 23: 281–291 8658212

[bib35] Offerhaus GJA, Giardiello FM, Krush AJ, Booker SV, Tersmette AC, Kelley NC, Hamilton SR (1992) The risk of upper gastrointestinal cancer in familial adenomatous polyposis. Gastroenterology 102: 1980–1982 1316858 10.1016/0016-5085(92)90322-p

[bib36] Oliveira C, Ferreira P, Nabais S, Campos L, Ferreira A, Cirnes L, Alves CC, Veiga I, Fragoso M, Regateiro F, Dias LM, Moreira H, Suriano G, Machado JC, Lopes C, Castedo S, Carneiro F, Seruca R. (2004) E-cadherin (CDH1) and TP53 rather than SMAD4 and caspase-10 germline mutations contribute to genetic predisposition in Portuguese gastric cancer patients. Eur J Cancer 40: 1897–1903 15288293 10.1016/j.ejca.2004.04.027

[bib37] Ottini L, Palli D, Falchetti M, D'Amico C, Amorosi A, Saieva C, Calzolari A, Cimoli F, Tatarelli C, De Marchis L, Masala G, Mariani-Costantini R, Cama A (1997) Microsatellite instability in gastric cancer is associated with tumor location and family history in a high risk population from Tuscany. Cancer Res 57: 4523–4529 9377564

[bib38] Palli D, Russo A, Ottini L, Masala G, Saieva C, Amorosi A, Cama A, D′Amico C, Falchetti M, Palmirotta R, Decarli A, Mariani Costantini R, Fraumeni Jr JF (2001) Red meat, family history, and increased risk of gastric cancer with microsatellite instability. Cancer Res 61: 5415–5419 11454685

[bib39] Parkin DM, Bray FI, Devesa SS (2001) Cancer burden in the year 2000. The global picture. Eur J Cancer 37(Suppl 8): S4–S66 11602373 10.1016/s0959-8049(01)00267-2

[bib40] Parkin DM (2004) International variation. Oncogene 23: 6329–6340 15322508 10.1038/sj.onc.1207726

[bib41] Renault B, Calistri D, Buonsanti G, Nanni O, Amadori D, Ranzani GN (1996) Microsatellite instability and mutations of p53 and TGF-beta RII genes in gastric cancer. Hum Genet 98: 601–607 8882883 10.1007/s004390050267

[bib42] Shimoyama S, Aoki F, Kawahara M, Yahagi N, Motoi T, Kuramoto S, Kaminishi M (2004) Early gastric cancer development in a familial adenomatous polyposis patient. Dig Dis Sci 49: 260–265 15104367 10.1023/b:ddas.0000017448.58196.dc

[bib43] Sicinschi LA, Lopez-Carrillo L, Camargo MC, Correa P, Sierra RA, Henry RR, Chen J, Zabaleta J, Piazuelo MB (2006) Gastric cancer risk in a Mexican population: role of *Helicobacter pylori* CagA positive infection and polymorphisms in interleukin-1 and -10 genes. Int J Cancer 118(3): 649–657 16114018 10.1002/ijc.21364

[bib44] Tsugane S, Sasazuki S (2007) Diet and the risk of gastric cancer: review of epidemiological evidence. Gastric Cancer 10: 75–83 17577615 10.1007/s10120-007-0420-0

[bib45] Wang P, Xia HH, Zhang JY, Dai LP, Xu XQ, Wang KJ (2007) Association of interleukin-1 gene polymorphisms with gastric cancer: a meta-analysis. Int J Cancer 120(3): 552–562 17096351 10.1002/ijc.22353

[bib46] Watson P, Lynch HT (1993) Extracolonic cancer in hereditary nonpolyposis colorectal cancer. Cancer 71: 677–685 8431847 10.1002/1097-0142(19930201)71:3<677::aid-cncr2820710305>3.0.co;2-#

[bib47] Yamada H, Shinmura K, Okudela K, Goto M, Suzuki M, Kuriki K, Tsuneyoshi T, Sugimura H (2007) Identification and characterization of a novel germ line p53 mutation in familial gastric cancer in the Japanese population. Carcinogenesis 28: 2013–2018 17690113 10.1093/carcin/bgm175

